# Phase II trial of concurrent chemoradiotherapy with L-asparaginase and MIDLE chemotherapy for newly diagnosed stage I/II extranodal NK/T-cell lymphoma, nasal type (CISL-1008)

**DOI:** 10.18632/oncotarget.11319

**Published:** 2016-08-16

**Authors:** Dok Hyun Yoon, Seok Jin Kim, Seong Hyun Jeong, Dong-Yeop Shin, Sung Hwa Bae, Junshik Hong, Seong Kyu Park, Ho-Young Yhim, Deok-Hwan Yang, Hyewon Lee, Hye Jin Kang, Mark Hong Lee, Hyeon-Seok Eom, Jae-Yong Kwak, Jae Hoon Lee, Cheolwon Suh, Won Seog Kim

**Affiliations:** ^1^ Department of Oncology, Asan Medical Center, University of Ulsan College of Medicine, Seoul, Korea; ^2^ Department of Medicine, Samsung Medical Center, Sungkyunkwan University School of Medicine, Seoul, Korea; ^3^ Department of Hematology-Oncology, Ajou University School of Medicine, Suwon, Korea; ^4^ Department of Internal Medicine, Seoul National University Hospital, Seoul, Korea; ^5^ Department of Internal Medicine, Catholic University of Daegu School of Medicine, Daegu, Korea; ^6^ Department of Internal Medicine, Gachon University Gil Medical Center, Incheon, Korea; ^7^ Department of Hematology/Oncology, Soonchunhyang University Bucheon Hospital, Bucheon, Korea; ^8^ Department of Internal Medicine, Chonbuk National University School of Medicine, Jeonju, Korea; ^9^ Department of Internal Medicine, Chonnam National University Medical School, Gwangju, Korea; ^10^ Center for Hematologic Malignancies, National Cancer Center, Goyang, Korea; ^11^ Department of Internal Medicine, Korea Cancer Center Hospital, Korea Institute of Radiological and Medical Sciences, Seoul, Korea; ^12^ Department of Hematology-Oncology, Konkuk University Medical Center, Seoul, Korea

**Keywords:** extranodal NK/T-cell lymphoma, nasal type, concurrent chemoradiotherapy, L-asparaginase, methotrexate, treatment

## Abstract

We designed a new treatment protocol incorporating concurrent administration of L-asparaginase (to reduce the probability of systemic progression during concurrent chemoradiotherapy (CCRT)) plus high-dose methotrexate to consolidation chemotherapy to intensify the regimen for treating localized extranodal NK/T cell lymphoma, nasal type (ENKTL). CCRT comprised radiation (36–44 Gy) with weekly cisplatin (30 mg/m^2^) and tri-weekly L-asparaginase (4 000 IU). Chemotherapy—MIDLE (methotrexate 3 g/m^2^ on day 1, etoposide 100 mg/m^2^ and Ifosfamide 1 000 mg/m^2^ on days 2–3, dexamethasone 40 mg on days 1–4, and L-asparaginase 6 000 IU/m^2^ on days 4, 6, 8, 10)—was repeated every 28 days for two cycles. One of the 28 patients developed distant lesions after CCRT. The final complete response rate was 82.1%. Four patients dropped out during or after their first MIDLE cycle due to toxicities (recurrent G3 hyperbilirubinemia [n = 1], G3-5 increased creatinine [n = 2], and G5 infection [n = 1]). With a median follow-up of 46 months (95% CI: 39–47 months), the estimated 3-year progression-free survival rate and overall survival rate were 74.1% and 81.5%, respectively. This MIDLE protocol may be effective for localized ENKTL. However, concurrent administration of L-asparaginase during CCRT does not seem to provide additional benefits.

## INTRODUCTION

Extranodal natural killer (NK)/T cell lymphoma (ENKTL) is a rare subtype of non-Hodgkin lymphoma (NHL) with a poor prognosis [1]. As the majority of ENKTL cases present as localized disease, particularly involving the nasal or paranasal area, treatment of localized disease has been an important issue [1, 2].

A Japanese group and our group have shown that concurrent chemoradiotherapy (CCRT) is a feasible and effective treatment for the management of localized ENKTL [2-4]. Furthermore, we have shown that CCRT with weekly cisplatin, followed by chemotherapy comprising etoposide, ifosfamide, cisplatin, and dexamethasone (VIPD) or etoposide, ifosfamide, dexamethasone, and L-asparaginase (VIDL) is an effective treatment strategy for the management of localized ENKTL [2, 3]. All the studies resulted in promising outcomes with 70–80% overall survival. Although prognosis in these patients has improved in recent years, the failure pattern shows that systemic failure predominates and disease progression occurs early in the majority [5]. Two patients (6.7%) among those enrolled in the phase 2 trial of VIDL also experienced systemic progression during CCRT, with development of new lesions in the liver or lung [2].

To further improve efficacy, we designed a new treatment protocol: methotrexate, ifosfamide, dexamethasone, L-asparaginase, and etoposide (MIDLE), which incorporates the tri-weekly administration of L-asparaginase (a key therapeutic agent for ENKTL) during CCRT to reduce the probability of systemic progression as well as high-dose methotrexate to intensify chemotherapy based on previous excellent outcomes of methotrexate-containing regimens such as dexamethasone, methotrexate, ifosfamide, L-asparaginase, etoposide (SMILE) and ifosfamide, methotrexate, L-asparaginase, prednisolone (IMEP) [6, 7].

## RESULTS

### Patient characteristics

Thirty patients with stage IE/IIE ENKTL were included this study from October 2010 to March 2012. Of these, two were excluded because of ineligibility (n = 1) and refusal (n = 1). The remaining 28 patients were included in the analysis. The median age at diagnosis was 51 years (range, 30–77 years). Circulating EBV DNA was detected in 4 patients (median 1.295 × 10^6^ copies/μL; range, 655–4.6 × 10^6^copies/μL), while it was less than the detectable level in 24 patients (Table 1). PINK-E scores were 0 in 16 patients and 1 in 12 patients.

**Table 1:** Sociodemographic and clinical characteristics of the studied patients

		Total (n = 28)	
Characteristics		Number	%
Age	Median 51, range: 30–77 years		
	>60	7	25.0
Sex	Male	24	85.7
Performance status	ECOG 0/1	28	100.0
Ann Arbor stage	IE	22	78.6
	IIE	6	21.4
Serum LDH	Increased	3	10.7
B symptoms	Presence	4	14.3
EBV in blood	Presence	4	14.3
Regional lymph node involvement	Presence	5	17.9
Primary site	Nasal cavity	25	89.3
	Nasopharynx or oropharynx	3	10.7
PINK-E	0	16	57.1
	1	12	42.9

Abbreviations: EBV, Epstein-Barr virus; IPI, international prognostic index; NKPI, NK/T cell lymphoma prognostic index; PINK-E, prognostic index for NK/T cell lymphoma-EBV.

### Toxicity and response to CCRT

All but two patients completed CCRT without interruption during treatment according to the protocol. One patient failed to finish CCRT following the protocol due to G3 mucositis with delayed recovery, and the other patient withdrew after G3 allergic reaction to L-asparaginase (Table 2). Three other patients stopped the administration of L-asparaginase during CCRT because of the development of repeated grade 2 allergic reactions to L-asparaginase, which was also not administered during MIDLE chemotherapy in those cases. Another two patients skipped their doses of cisplatin and L-asparaginase due to grade 3 nausea/vomiting and grade 2 azotemia, respectively.

**Table 2:** Sociodemographic and clinical characteristics of the studied patients

Toxicity	CCRT (n = 28)				MIDLE (n = 23)			
	G1	G2	G3	G4	G1	G2	G3	G4
Hematologic								
Anemia	3	2			2	3	2	
Neutropenia		1	2				2	19
Thrombocytopenia						3	1	2
Febrile neutropenia					1	1	10	
Non-hematologic								
Nausea	4	3	11		4	2	6	
Vomiting	5	3	3		3	2		
Diarrhea	1	1			1	3		
Anorexia	2	1	5		6	3	4	
Constipation	5	1			2	1		
Stomatitis	4	8	1		6	6	2	
General weakness	2		1		3	1	1	
Insomnia	1	1			1	1		
Allergic reaction	2	3	1		2	2	2	
Alopecia	3				3	3		
Infection								1 (G5)*
Creatinine elevation		2			1	1	1	1 (G5)*
Amylase elevation	2							
Transaminase elevation	6	6	1		2	3	3	
Bilirubin elevation	1	8	3		1	3	1	

* A 51-year-old man died of acute kidney injury complicated by pneumonia with sepsis on C1D19 after MIDLE chemotherapy.

Sixteen patients (57.1%) achieved CR and 8 a partial response (PR), with an overall response rate of 85.7%. One patient showed stable disease (SD) after CCRT. The other patient showed progressive disease (PD) with development of new lesions in the retroperitoneal lymph node and spleen. This patient had the highest level of EBV (4.6 × 10^6^ copies/μL) at baseline. After completion of CCRT, two patients in CR after CCRT could not proceed to MIDLE chemotherapy treatment due to poor performance (n = 1) and decreased renal function (n = 1).

### Toxicity and response to MIDLE chemotherapy

Twenty-three patients received MIDLE chemotherapy and 20 of those completed the planned 2 cycles of chemotherapy. However, MIDLE chemotherapy was associated with high rate of grade 3 or 4 neutropenia (91.3%, n = 21) and febrile neutropenia (43.5%, n = 10) (Table 2). Two patients, each on C1D2 (G3) and C1D3 (G5), experienced acute kidney injury and they could not proceed to the second cycle. One of them died of pneumonia complicated by sepsis after development of acute kidney injury. Another one was dropped from this study due to recurrent grade 3 bilirubinemia during cycle 1. One patient required 25% dose reduction of methotrexate and skipped two doses of L-asparaginase during cycle 2 due to G3 mucositis. However, no patient had L-asparaginase-associated acute pancreatitis or coagulopathy during or after MIDLE chemotherapy. Administration of L-asparaginase was stopped in 2 patients due to grade 3 allergic reactions.

All 20 patients those who completed the 2 cycles of MIDLE chemotherapy achieved CR including those 6 patients with PR and 1 SD after CCRT. Therefore, the final CR rate was 82.1 % (23 out of 28) after CCRT, followed by MIDLE chemotherapy.

### Survival outcomes and prognostic indices

**Figure 1:**
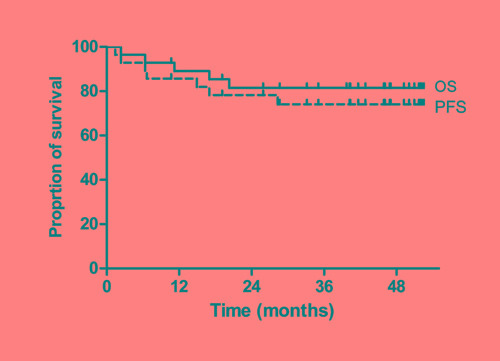
Progression-free survival (PFS) and overall survival (OS)

The last survival data update was performed in July 2015. With a median follow-up of 46 months (95% confidence interval: 39–47 months), four patients progressed and five patients died with the estimated 3-year PFS rate of 74.1% and OS rate of 81.5%, respectively (Figure 1).

A 50-year old man with detectable EBV in blood experienced local recurrence in the nasal cavity at 19.5 months and underwent retrial of CCRT followed by SMILE chemotherapy and high-dose chemotherapy with autologous stem cell transplantation (HDT-ASCT). The other three patients developed systemic progressive diseases. All of them were young (<60 years), and EBV was detected in two of them at diagnosis. In other words, 3 of 4 patients (with EBV positivity in blood) experienced disease progression in contrast to only 1 of 24 with EBV negativity. One of the patients (who had disease progression at 28 months) was salvaged with SMILE and HDT-ASCT and survived, but the other two with disease progression just after CCRT (PFS 1.4 months) or MIDLE (PFS 6.4 months) died despite receiving salvage chemotherapy such as SMILE or IMEP. In addition to the two deaths due to PD, a 51-year old man died of pneumonia with sepsis complicated by acute kidney injury during MIDLE chemotherapy (as described above) and two additional elderly patients in CR (aged 72 and 77 years, respectively) died of pneumonia during follow-up; one died at 1 year after MIDLE chemotherapy and the other after 3.5 months.

Patients with a zero PINK-E score tended to have better PFS (3-year PFS rate of 87.5% vs. 54.0%, p = 0.078) and OS (3-year OS rate of 87.5% vs. 73.3%, p = 0.414) although those observations were not statistically significant (Supplementary Figure 1).

## DISCUSSION

The aim of this trial was to develop a more effective treatment strategy with the incorporation of L-asparaginase in addition to weekly cisplatin during CCRT (to lower the risk of early systemic treatment failure) along with the addition of high-dose methotrexate to the VIDL-like chemotherapy with modifications including reduced dose of both etoposide and ifosfamide [2]. This treatment resulted in a 82.1% CR rate with an estimated 3-year PFS rate of 74.1% and OS rate of 81.5%.

All patients in PR or SD after CCRT could achieve CR after two cycles of MIDLE chemotherapy. Efficacies seem comparable to those from prior trials of VIPD, VIDL, and DeVIC; CR rates ranging from 77% to 87%, 2- to 5-year PFS rates 67% to 85.2%, and 2- to 5-year OS rates 60% to 86.3% (Table 3) [2-4].

**Table 3:** Regimens used for localized extranodal NK/T cell lymphoma

	MIDLE (n = 28)	VIPD (n = 30)[3]	VIDL (n = 30)[2]	DeVIC (n = 27)[4]	SMILE* (n = 38)[6]
**Radiotherapy**	36–44 Gy	40–52.8 Gy	40–44 Gy	50 Gy	-
**Regimens for CCRT**	Weekly cisplatin 30 mg/m^2^				-
	+ triweekly L-asp 4 000	Weekly cisplatin 30 mg/m^2^	Weekly cisplatin 30 mg/m^2^	DeVIC	
	IU/m^2^				
**Chemotherapy**					-
Methotrexate	3 g/m^2^ on D1	-	-	-	2 g/m^2^ on D1
Epotoside	100 mg/m^2^ on D2-3	100 mg/m^2^ on D1–3	100 mg/m^2^ on D1–3	67 mg/m^2^ on D1–3	100 mg/m^2^ on D2–4
Ifosfamide	1 000 mg/m^2^ on D2-3	1 200 mg/m^2^ on D1–3	1 200 mg/m^2^ on D1–3	1 000 mg/m^2^ on D1–3	1 500 mg/m^2^ on D2–4
Platinum	–	Cisplatin	–	Carboplatin	–
		33 mg/m^2^ on D1–3		200 mg/m^2^ on D1	
Dexamethasone	40 mg/day on D1–4	40 mg/day on D1–4	40 mg/day on D1–4	40 mg/day on D1–3	40 mg/day on D2–4
L-asparaginase	6 000 IU/m^2^, 4 doses		4 000 IU/m^2^, 7 doses		6 000 IU/m^2^, 7 doses
No of cycles	2 cycles	3 cycles	2 cycles	3 cycles	2 cycles
	every 4 weeks	every 3 weeks	every 4 weeks	every 3 weeks	Every 28 days
**CR rate**	82.1%	80.0%	87%	77%	45%*
**PFS**	3-yr PFS, 74.1%	3-yr PFS, 85.2%	5-yr PFS, 73%	2-yr PFS, 67%	1-yr PFS, 53%
**OS**	3-yr OS, 81.5%	3-yr OS, 86.3%	5-yr OS, 60%	2-yr OS, 78%	1-yr OS, 55%
**G3-4 neutropenia**	91.3%	46.7%	80%	90.9%	100%
**G3-4 FN**	43.5%	60%	16.7%	18.2%	NA
**TRM**	1; AKI and pneumonia	2; infection	0	0	2 (infection)

* Phase 2 trial of SMILE involved stage 4, relapsed or refractory extranodal NK/T cell lymphoma unlike the other trials involving localized stage disease.

Abbreviations: AKI, acute kidney injury; CCRT, concurrent chemoradiotherapy; CR, complete response; D1, day 1. FN, febrile neutropenia; NA, not available; OS, overall survival; PFS, progression-free survival; TRM, treatment-related mortality.

Although we aimed to reduce the probability of systemic failure during CCRT, one patient (3.6%) developed new lesions in the distant lymph node and spleen just after CCRT. Concurrent administration of L-asparaginase during CCRT does not seem to provide additional benefit, although we cannot make definite conclusions comparing this failure rate with prior trials including VIPD (0 of 30) and VIDL (2 out of 30) which utilized just weekly cisplatin during CCRT. However, six patients experienced hypersensitivity to L-asparaginase during chemoradiotherapy resulting in skipping of doses or discontinuation of L-asparaginase. In addition, administration of L-asparaginase during MIDLE chemotherapy was stopped in two additional patients due to the allergic reactions.

Clinical hypersensitivity to native *Escherichia coli* L-asparaginase has been reported to range from 32.5% to 75% [8]. In the SMILE chemotherapy, 5 out of 38 patients experienced grade 1 to 3 allergic reactions to L-asparaginase, which was not observed during VIDL. Grade 3 or 4 gastrointestinal toxicity, including nausea (n = 11), vomiting (n = 3), anorexia (n = 5), and cholestatic injury with hyperbilirubinemia (n = 3), was also relatively common compared with prior studies during CCRT. Thus, L-asparaginase should be administered with caution and vigilant monitoring of possible adverse events is advised during concurrent chemoradiotherapy.

Although the frequency of hematologic toxicities—including grade 3 or 4 neutropenia (n = 21, 91.3%) and febrile neutropenia (n = 10, 43.5%)—does not seem to be higher compared with those of prior trials (G3 or 4 neutropenia: 46.7%–91.3%; G3 or 4 febrile neutropenia: 16.7%–60%; Table 3); those rates seem to be still significantly high considering the generally fair prognosis of the localized ENKTL [2-4]. In addition, two patients experienced acute kidney injury on day 2 or 3 of the first cycle of MIDLE and one of them died of pneumonia complicated by sepsis, which seems to be related to high-dose methotrexate.

Nephrotoxicity is also a significant toxicity in SMILE (containing 2.0 g/m^2 ^methotrexate); 5% experienced grade 3 azotemia in the phase 2 trial and 17.2% (including one acute kidney injury and death) experienced grade 3 azotemia in a study from the Asia Lymphoma Study Group [6, 9]. These risks of renal failure in patients with ENKTL seem too high compared with the 2% recorded in children treated with high-dose methotrexate for osteosarcoma [10]. Precautions with strict prophylactic measures including alkaline hydration must therefore be taken with such regimens containing high-dose methotrexate.

Recently we proposed both PINK and PINK-E as prognostic models of ENKTL in nonanthracycline-based treatment [11]. However, PINK must have a limitation in discriminating prognosis in this early-stage nasal-type disease because all the patients have stage 1 or 2 disease, nasal involvement, and no distant lymph node involvement therefore leaving just age as a valid variable for this model. In the case of PINK-E, both age and EBV-PCR would be effective predictors among five variables resulting in classification of elderly patients with EBV-PCR positivity as an intermediate-risk group. Although no enrolled patients were classified as higher-risk due to their small number, those with PINK-E score 1 still had a tendency for lower PFS and OS rates compared with those with PINK-E score 0.

In conclusion, L-asparaginase plus CCRT followed by MIDLE chemotherapy may be an effective treatment strategy for stage I/II extranodal NK/T-Cell lymphoma, nasal type. However, compared with a previous study, higher numbers of patients were discontinued during or after CCRT due to toxicity or poor tolerance. Concurrent administration of *E. coli* L-asparaginase during CCRT does not seem to provide additional benefits. Precautions should be taken when a high-dose methotrexate-containing regimen is given for early-stage disease due to possible adverse effects.

## MATERIALS AND METHODS

### Patients

Eligibility criteria included newly diagnosed ENKTL based on the presence of histological features and immunohistochemistry results, including cytoplasmic CD3+, CD20−, and CD56+ positive for cytotoxic molecules and Epstein–Barr virus (EBV) in *in situ* hybridization. None of the patients received any treatment for ENKTL, and all had measurable disease.

Patients were 18 years of age or older, and their disease state was Ann Arbor stage IE or IIE. Additional eligibility criteria were as follows: Eastern Cooperative Oncology Group (ECOG) performance status of 0–2; hemoglobin ≥ 9.0 g/dL; absolute neutrophil count ≥ 1500/μL and platelet count ≥ 100 000/μL; serum creatinine levels ≤ 1.5 mg/dL and creatinine clearance ≥ 50 mL/min; total bilirubin < two times the upper limit of normal; and aspartate transferase < three times the upper limit of the normal value.

Patients who had any coexisting medical diseases with sufficient severity to prevent full compliance with the study protocol such as heart failure or acute, active infection were excluded from the study. Considering the adverse effects of L-asparaginase, patients who had a history of acute pancreatitis were also excluded.

### Study design and objectives

CCRT consisted of radiation therapy with 36–44 Gy per 18–22 fractions and weekly administration of cisplatin (30 mg/m^2^) and tri-weekly intravenous or intramuscular L-asparaginase (4 000 IU/m^2^) on days 1,3, and 5 in each week during radiotherapy. All patients received three-dimensional conformal radiotherapy or intensity-modulated radiotherapy. The clinical target volume was delineated with adequate margins surrounding the gross target volume, considering the anatomic boundary of the involved subsite. The addition of elective lymphatic irradiation was determined on an individual basis [3, 12]. The first response evaluation was performed 3 to 4 weeks after the completion of CCRT.

Two cycles of MIDLE chemotherapy (methotrexate 3 g/m^2^ on day 1, intravenous administration of etoposide (100 mg/m^2^), ifosfamide (1 000 mg/m^2^), and dexamethasone (40 mg) on days 2–4, followed by intravenous or intramuscular injection of L-asparaginase (4 000 IU/m^2 ^on days 4, 6, 8, and 10) was repeated every 4 weeks. The final response evaluation was performed 6 to 8 weeks after the completion of the second cycle of MIDLE. The primary endpoint was complete response (CR) rate, including CR-unconfirmed (CR-u) by investigator review; and the secondary objectives included overall response rate, overall survival (OS), progression-free survival (PFS), and toxicities.

All patients provided written informed consent. The study was reviewed and approved by the institutional review board at each participating institute and was registered at ClinicalTrials.gov (NCT01238159).

### Assessment of response and toxicity

Baseline assessments included complete blood count, determination of serum lactate dehydrogenase (LDH) levels, bone marrow aspiration and trephine biopsy, endoscopic examination of the nasal and oral cavities by otorhinolaryngologists, CT scanning or magnetic resonance imaging (MRI) of the involved lesions, CT scanning of the chest and abdomen–pelvis, and ^18^F-fluorodeoxyglucose positron emission tomography (FDG-PET).

All these studies were performed before treatment and after completion of CCRT and MIDLE, and, to monitor relapse, they (except FDG-PET) were then repeated every 6 months thereafter for 2 years and yearly thereafter for the following 3 years. Subsequently, survival status was monitored at each participating institute, and evaluation of disease status to monitor relapse was performed at the physicians’ discretion.

For prognostic factor analysis, prognostic index for NK/T cell lymphoma-EBV (PINK-E) was evaluated [11]. Quantitative polymerase chain reaction of EBV DNA in the peripheral blood was also performed as proposed previously [13, 14]. The treatment response was assessed according to the International Working Group response criteria [15]. Toxicity was evaluated before each treatment cycle according to the National Cancer Institute Common Toxicity Criteria (NCI CTC) version 4.0. Dose modification of cisplatin during CCRT as well as that of etoposide and ifosfamide was performed as reported previously [3].

If patients experienced grade 1 or 2 allergic reactions or hypersensitivity to L-asparaginase, treatment was interrupted until the toxicity resolved and treatment was resumed with a 50% dose reduction; however, it was omitted if the toxicity repeated. If the patients had grade 3 or 4 allergic reactions or hypersensitivity, pancreatitis, or hypotension, then L-asparaginase was omitted.

### Statistics

The sample size was determined based on CR rates according to Simon's optimal two-stage design [17] and assuming a target and a lower activity level of 0.90 (p1) and 0.70 (p0), respectively. The minimum target number of subjects was 27 in total. This design provided a probability of 0.05 of accepting a treatment worse than p0 and a probability of 0.20 for rejecting a treatment better than p1. If we assume that the dropout rate is 10%, the total accrual needed to be 30 patients.

The association between patient characteristics and treatment response was analyzed using the Chi-square test. The Kaplan–Meier method was used to calculate PFS and OS, and survival curves were compared via the log-rank test. A two-sided P value <0.05 was considered significant. PFS was defined as the time from the date of enrollment to the date of documented disease progression or any kind of death. OS was measured from the date of enrollment to the date of death from any cause and was censored at the date of the last follow-up visit.

## SUPPLEMENTARY MATERIALS FIGURES AND TABLES


