# Supplementary material for: Phase II trial of concurrent chemoradiotherapy with L-asparaginase and MIDLE chemotherapy for newly diagnosed stage I/II extranodal NK/T-cell lymphoma, nasal type (CISL-1008)
Source: Oncotarget. 2016 Aug 16;7(51):85584–91. doi: 10.18632/oncotarget.11319 (PMC5356760; doi:10.18632/oncotarget.11319)
Supplement: Supplementary file 1 [file oncotarget-07-85584-s001.pdf]

Phase II trial of concurrent chemoradiotherapy with L-asparaginase and MIDLE chemotherapy for newly diagnosed stage I/II extranodal NK/T-cell lymphoma, nasal type (CISL-1008)

Supplementary Material

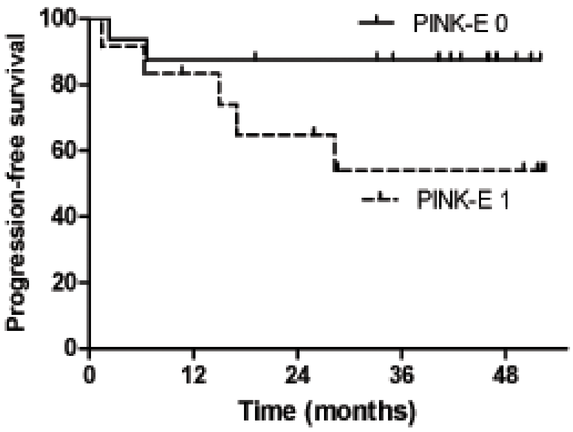

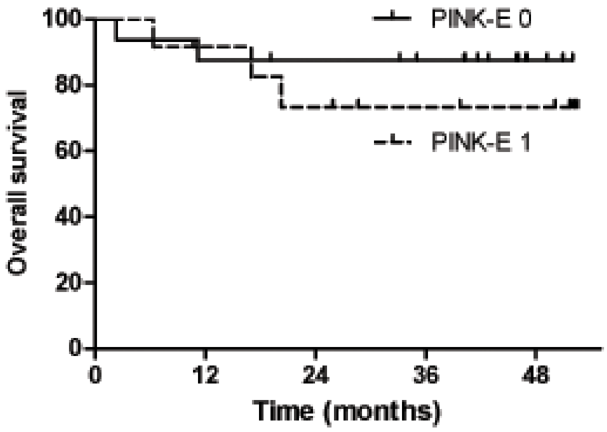

Supplementary Figure 1. Patients with a PINK-E score of 1 tend to have inferior PFS (p = 0.078) and OS (p = 0.414) in localized extranodal NK/T cell lymphoma, nasal type.

(A) PFS by PINK-E

(B) OS by PINK-E
